# FGF2-induced PI3K/Akt signaling evokes greater proliferation and adipogenic differentiation of human adipose stem cells from breast than from abdomen or thigh

**DOI:** 10.18632/aging.103547

**Published:** 2020-07-24

**Authors:** Guan-Ming Lu, Yong-Xian Rong, Zhi-Jie Liang, Dong-Lin Hunag, Fang-Xiao Wu, Yan-Fei Ma, Zhi-Zhai Luo, Xin-Heng Liu, Steven Mo, Hong-Mian Li

**Affiliations:** 1 Department of Breast and Thyroid Surgery, Affiliated Hospital of Youjiang Medical University for Nationalities, Guangxi 533000, China; 2 Department of Burn and Plastic Surgery, Guiping People’s Hospital, Guigping 537200, Guangxi, China; 3 Department of Plastic and Aesthetic Surgery, The Fifth Affiliated Hospital of Guangxi Medical University and The First People’s Hospital of Nanning, Nanning 530022, Guangxi, China; 4 Nanning Life-Ontology Biotechnology Co., Ltd., Nanning 530229, Guangxi, China

**Keywords:** adipose-derived stem cells, molecular signature, paracrine, adipogenic differentiation, depot-specific stem cell populations

## Abstract

In this study, human adipose stem cells were isolated from subcutaneous fat in the thigh (htASCs), abdomen (haASCs) and breast (hbASCs). Flow cytometry was used to detect cell surface markers, and an enzyme-linked immunosorbent assay was used to detect paracrine activity. Paracrine gene expression in the three cell types was examined using real-time qPCR, and adipogenic ability was assessed using Oil Red O staining. RNA from third-passage haASCs and hbASCs was sequenced. The results showed that the differentiation potential marker markers CD49d and CD54 were similar across hbASCs from 10 subjects. The hbASCs showed higher colony forming ability and expression of fibroblast growth factor-2, tissue inhibitor of metalloproteinase-1 and stromal cell derived factor-1 than htASCs and haASCs. Stimulating hbASCs with FGF2 promoted adipogenic differentiation, while treating the cells with the PI3K inhibitor LY294002 inhibited differentiation. These results suggest that the PI3K/Akt signaling pathway can promote proliferation and adipogenic differentiation of adipose stem cells, and that activation of this pathway by FGF2 may explain why hbASCs show greater proliferation and adipogenic differentiation than haASCs and htASCs.

## INTRODUCTION

Over the last decade, autogenous fat transplantation, reconstruction of soft tissue injury and cosmetic breast augmentation have received greater attention and found wider use [1–4]. However, the safety and effectiveness of autogenous fat transplantation remain the focus of debate. Failure of autogenous fat transplantation may lead to fat necrosis, microcalcification and inflammation of cyst formation [5–8]. Recent advances in regenerative medicine have put adipose stem cells (ASCs) in the spotlight for their paracrine activity [9], differentiation ability and potential application in tissue engineering. Many studies have shown that ASCs show excellent regenerative potential for fat transplantation in the clinic [10–12].

ASCs, easily obtained from adipose tissue, are a subset of mesenchymal stem cells (MSCs) showing similar regeneration characteristics as other MSCs [13–15]. ASCs easily adhere to plastic culture bottles and expand *in vitro* and show the potential to repair, maintain or enhance various tissues [16]. Many studies have demonstrated that ASCs are capable of differentiating into mesenchymal and non-mesenchymal cell types *in vitro* and *in vivo,* including adipocytes, chondrocytes, osteoblasts, neurons, myocytes, and endothelial cells [17–21]. Moreover, ASCs can promote tissue regeneration by secreting cytokines and growth factors, thus promoting the recovery of normal tissue function or reducing tissue damage [22]. These favorable characteristics of human adipose tissue offer a practical, alternative source of MSCs for use in regenerative medicine. However, ASCs harvested from different anatomical areas exhibit different characteristics [23, 24]. This suggests that there may be an optimal source of ASCs for autologous transplantation.

In this study, we isolated ASCs from human thigh (htASCs), abdomen (haASCs) and breast (hbASCs) and compared their molecular characteristics and ability to differentiate into adipocytes and develop paracrine function *in vitro* and *in vivo*. Our intention was to identify the best source of ASCs for potential clinical use. Moreover, we combined cell biology and bioinformatics to explore what mechanisms may help explain differences in proliferation and differentiation among ASCs from different sources.

## RESULTS

### Characteristics of ASCs from three tissue sources

**Figure 1:**
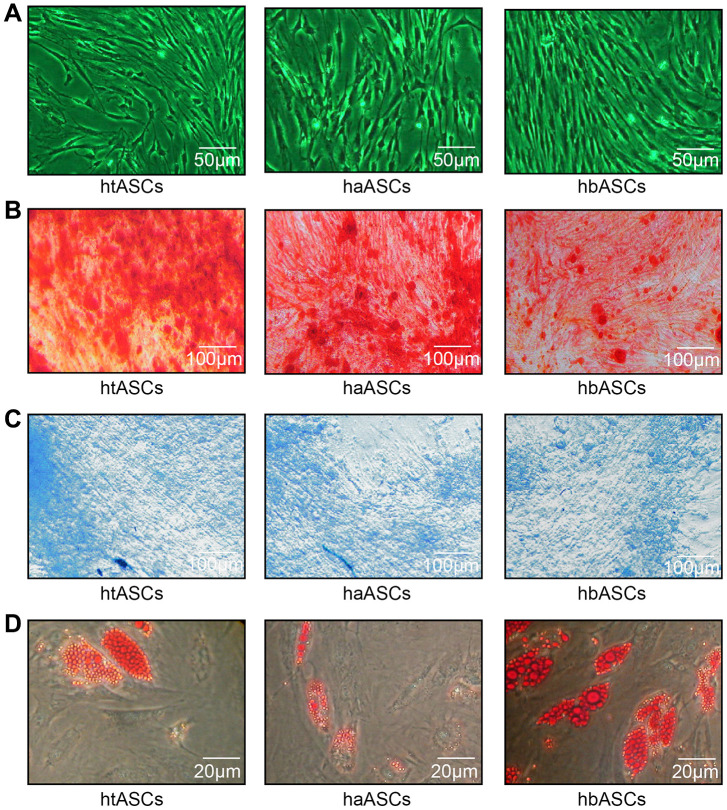
**Proliferation and differentiation of haASCs, htASCs and hbASCs.** (**A**) Passage 3 (P3) of htASCs, haASCs, and hbASCs. (**B**) Positive Alizarin Red staining of P3-htASCs, P3-haASCs, and P3-hbASCs following 3 weeks of osteogenic induction. (**C**) Positive Alcian Blue staining of P3-htASCs, P3-haASCs, and P3-hbASCs following 2 weeks of chondrogenic induction. (**D**) Positive Oil Red O staining of P3-htASCs, P3-haASCs, and P3-hbASCs following 2 weeks of adipogenic induction.

The htASCs, haASCs, and hbASCs began to adhere to the plates within 6 h after seeding. Initially, the cells were small, round, and irregular in size, with some mononuclear blood cells. By 48 h post-seeding, the cells gradually stretched into short or long spindle shapes with fibroblast-like morphology. Cells achieved 80-90% confluence after 7-8 days. After passage, all three cell types displayed typical fibroblast-like morphology; during subculture, cells reached the same confluence within 3-4 days after passage, with a 1:3 split ratio at P3 (Figure 1A). P3 ASCs were cultured with osteogenic, chondrogenic, or adipogenic induction medium, and the corresponding lineage-specific cell morphologies were observed after 2, 3, or 2 weeks of culture, respectively, based on staining with Alizarin Red, Alcian Blue, or Oil Red O. All three ASCs were able to undergo osteogenesis, chondrogenesis and adipogenic differentiation (Figure 1B–1D).

We further explored the immunophenotypic characteristics of ASCs, and the results confirmed the multipotency of htASCs, haASCs, and hbASCs. Flow cytometry showed that at P1, htASCs, haASCs, and hbASCs were positive for expression of mesenchymal surface markers HLA-ABC, CD13, CD29, CD44, CD49d, CD54, CD90, and CD105, and negative for expression of surface markers HLA-DR, CD14, CD31, CD34, CD45, and CD106. Expression of CD49d and CD54 was significantly higher in hbASCs than in htASCs or haASCs, whereas the three groups did not differ significantly in the expression of HLA-ABC, CD13, CD29, CD44, CD90, or CD105 (Table 1).

**Table 1:** Expression of various markers in undifferentiated ASCs at P1 by flow cytometric analysis (%).

**Marker**	**htASCs(n=3)**	**haASCs(n=3)**	**hbASCs(n=4)**
**HLA-ABC**	93.6±3.2	90.5±2.8	92.9±3.3
**HLA-DR**	1.9±0.3	2.1±0.6	1.7±0.4
**CD13**	99.1±0.4	97.7±0.8	98.8±0.5
**CD14**	2.3±0.4	2.7±0.5	2.6±0.5
**CD29**	98.5±1.2	95.8±1.1	97.6±1.4
**CD31**	1.2±0.5	1.7±0.6	1.5±0.7
**CD34**	4.7±0.7	4.5±0.6	4.2±0.5
**CD44**	91.3±2.5	93.4±2.9	92.2±2.6
**CD45**	3.9±0.8	4.2±0.9	3.8±0.7
**CD49d**	36.7±1.8	34.8±2.2	58.6±3.1^*^
**CD54**	29.4±1.6	31.6±1.9	43.5±2.7^*^
**CD90**	88.6±2.3	90.2±3.3	87.5±2.8
**CD105**	90.5±2.6	93.1±3.4	91.4±2.9
**CD106**	2.7±0.9	3.3±0.9	3.1±0.8

* P<0.05 vs htASCs or haASCs.

### Proliferation and differentiation differences among haASCs, htASCs and hbASCs *in vitro*

**Figure 2:**
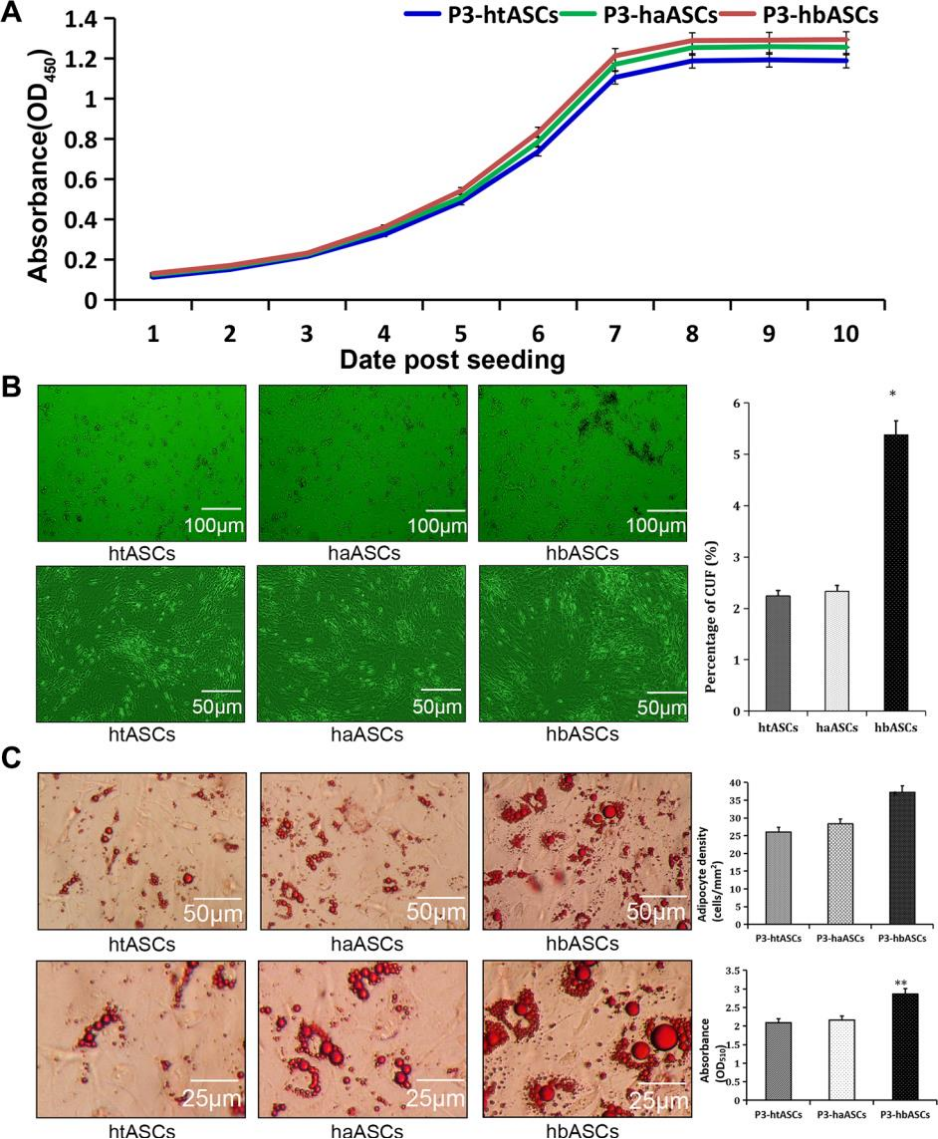
**Different proliferation and differentiation abilities of haASCs, htASCs and hbASCs *in vitro.*** (**A**) Cell proliferation as assessed by a CCK-8 assay. The proliferation rate and growth capacity of htASCs were similar to those of haASCs or hbASCs, but there was no significant difference in cell doubling time among htASCs, haASCs, and hbASCs. (**B**) Colony formation assays. Cells within the colonies exhibited long-spindle, short-spindle, short-round, or long-narrow morphology. After 14 days, colony forming units (CFUs) were significantly higher for hbASCs than for htASCs or haASCs (P < 0.05). (**C**) Adipogenic differentiation assay of htASCs, haASCs, and hbASCs *in vitro*. Adipocyte density and lipid concentration were significantly different between hbASCs and the other two ASC types. *P<0.05, **P<0.05

During the normal culture process, CCK-8 tests were performed on the htASCs, haASCs, and hbASCs. Proliferation rate and growth capacity were similar among these three types of ASCs (Figure 2A), with no significant difference in growth ability or cell doubling time, which was 39.13±4.30 min for htASCs, 40.63±2.97 min for haASCs, and 40.16±1.69 min for hbASCs.

Colony formation was also calculated for htASCs, haASCs, and hbASCs. After 10 days of culture, single cell-derived colonies were observed, consisting of 100-150 cells. Cells within the colonies were classified as long spindle, short spindle, short round, or long narrow shaped (Figure 2B). The hbASC colonies grew gradually, while colonies of htASCs and haASCs grew slowly. Results revealed significantly greater colony formation by hbASCs (5.38%±0.27%) than htASCs (2.24%±0.17%, P < 0.05) and haASCs (2.33%±0.19%, P < 0.05) (Figure 2B).

Moreover, we found that htASCs, haASCs, and hbASCs exhibited different adipogenic potential *in vitro*. After 14 days of induction of adipogenic differentiation, hbASCs contained a large number of Oil Red O-positive lipid droplets in their cytoplasm. In contrast, htASCs and haASCs contained relatively few such droplets (Figure 2C). The hbASCs also showed higher adipocyte density and lipid concentration than htASCs and haASCs.

### Different proliferation and adipogenic differentiation abilities of haASCs, htASCs and hbASCs *in vivo*

**Figure 3:**
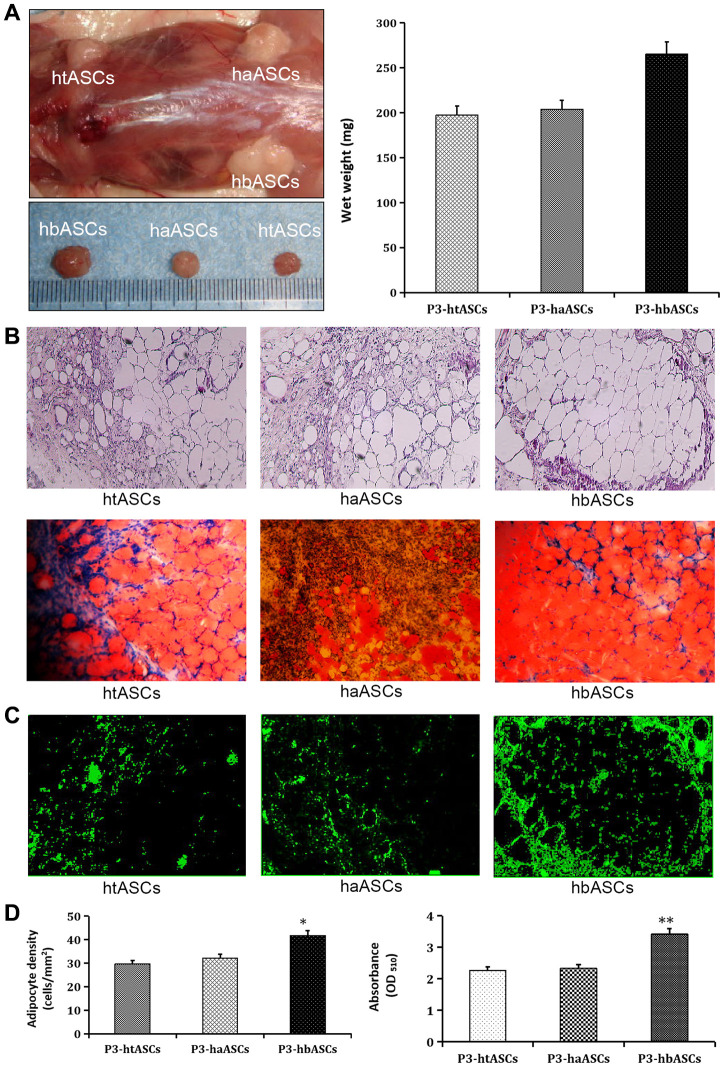
**Different proliferation and differentiation abilities of haASCs, htASCs and hbASCs *in vivo.*** (**A**) Regenerative adipose tissue macroscopic findings. Wet weights of regenerative adipose tissue in htASCs, haASCs, and hbASCs. *P<0.05. (**B**) H&E staining of the regenerative tissue after 12 weeks. The transplants derived from the three ASC types consisted predominantly of mature adipose tissue. Magnification, 100×. (**C**) GFP staining of the regenerative tissue after 12 weeks. In contrast to htASC or haASC tissue, GFP+ hbASC tissue contained larger Oil Red O-positive lipid droplets in the cytoplasm. GFP+ cells were detected in regenerative mature adipose tissue, indicating that these mature adipocytes had differentiated from GFP-labeled ASCs. Magnification, 100×. (**D**) Quantitative measurement of adipogenesis ability. Adipocyte density and intracellular lipid content were higher in hbASC tissue than in htASC or haASC tissue. *P < 0.05,**P < 0.05.

All animals were assessed at 12 weeks after transplantation. Macroscopic findings demonstrated that regenerated tissue had formed at the implantation site, and this tissue was excised for subsequent testing. The wet weight of the neogenic tissue was significantly greater in hbASCs than in htASCs or haASCs (Figure 3A). H&E staining indicated that the regenerated tissue in all three groups was composed of adipose tissue that had matured to differing degrees (Figure 3B): the tissue in the case of hbASCs was more mature and showed no signs of fibrosis. The collagen sponges degraded during the 12-week interval since implantation.

Neogenic tissue was also visualized by GFP (Figure 3C), indicating that the mature adipose cells had differentiated from GFP-labeled ASCs. Oil Red O staining showed that neogenic tissue derived from hbASCs contained more Oil Red O-positive lipid droplets within the cytoplasm, more adipocytes and higher intracellular lipid content than tissue derived from htASCs or haASCs (Figure 3D). These data indicate differing adipogenic potential in the various ASCs.

### DEGs between haASCs and hbASCs

**Figure 4:**
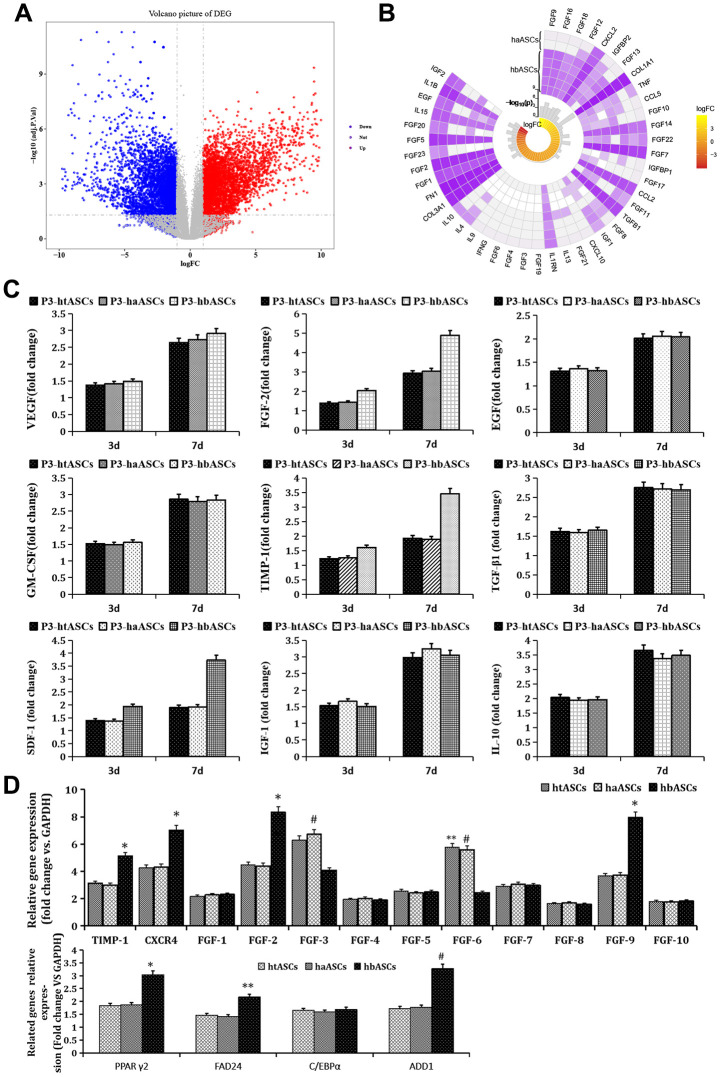
**Genes differentially expressed between haASCs and hbASCs.** (**A**) Volcano map. Red means up-regulated genes; blue means down-regulated genes. (**B**) Circplot associated with paracrine genes of ASCs. (**C**) ELISA of paracrine factors produced by htASCs, haASCs, and hbASCs *in vitro*. Higher levels of FGF2, TIMP-1, and SDF-1 were secreted by hbASCs than by htASCs or haASCs at 3 and 7 d. *P<0.05. (**D**) Differences in expression of genes related to paracrine function and adipogenesis across the three types of transplants. The hbASC tissue showed up-regulation of TIMP-1, CXCR4, FGF2, FGF9, PPAγ2, FAD24 and ADD1, but down-regulation of FGF3 and FGF6 relative to htASC and haASC tissue. C/EBPα expression was similar between htASC and haASC tissue. *P < 0.05, **P < 0.05, #P < 0.05

Of 12,466 DEGs between haASCs and hbASCs, 5,699 were up-regulated and 6,769 down-regulated in hbASCs (Figure 4A). These genes included several involved in paracrine function: FGF9, FGF16, FGF18, FGF12, IGFBP2, CXCL2, FGF13, CCL5 and FGF10 were up-regulated in hbASCs, while IGF2, IL1B, FGF20, FGF5, EGF, IL15, FGF23 and FGF2 were down-regulated (Figure 4B).

At days 3 and 7 of cell culture, concentrations of FGF2, TIMP-1, and SDF-1 in culture medium were higher in hbASCs than in htASCs or haASCs (Figure 4C). Moreover, qPCR analysis demonstrated that levels of mRNAs encoding TIMP-1, CXCR4, FGF2, or FGF9 were significantly higher, while levels of mRNAs encoding FGF3 and FGF6 were significantly lower, in neogenic tissue derived from hbASCs than in tissue derived from htASC or haASC (Figure 4D). Expression of FGF1, FGF4, FGF5, FGF7, FGF8, and FGF10 was similarly low across all three groups.

Analysis of adipogenesis-related genes revealed that PPARγ2, FAD24, and ADD1 were expressed at higher levels in hbASCs than in htASCs and haASCs (Figure 4D). However, C/EBPα expression was similar across neogenic tissue samples from the three groups.

### BPs and pathways involving DEGs

**Figure 5:**
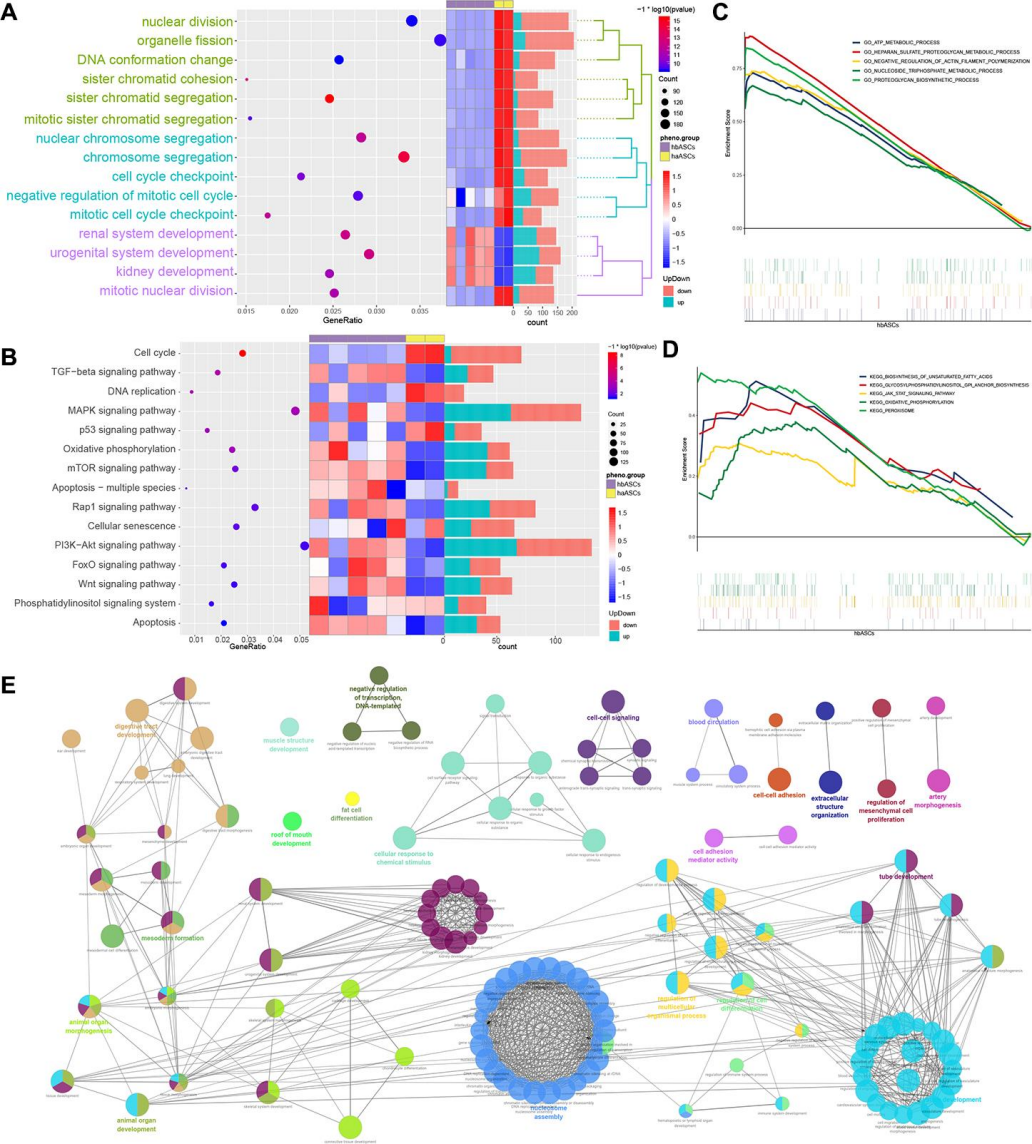
**Analysis of enrichment in biological processes and KEGG pathways.** (**A**) Biological process enrichment. (**B**) KEGG pathway enrichment. (**C**) GSEA of biological processes. (**D**) GSEA of KEGG pathways. (**E**) ClueGO analysis of biological processes.

Enrichment analysis showed that DEGs were significantly involved in BPs such as nuclear division, organelle fission, DNA conformational change, sister chromatid segregation, nuclear chromosome segregation, chromosome segregation, negative regulation of the mitotic cell cycle, renal system development and urogenital system development (Figure 5A). Moreover, DEGs were significantly involved in KEGG pathways such as as the cell cycle, MAPK signaling pathway, mTOR signaling pathway, cellular senescence and PI3K-Akt signaling pathway (Figure 5B).

GSEA showed that hbASCs were enriched in genes involved in ATP metabolism, HEparan sulfate proteoglycan metabolism, negative regulation of actin filament polymerization, nucleoside triphosphate metabolism and proteoglycan biosynthesis (Figure 5C, 5D). These genes were also involved in biosynthesis of unsaturated fatty acids, glycosylphosphatidylinositol anchor biosynthesis, JAK-STAT signaling, oxidative phosphorylation and peroxisome assembly and function.

ClueGO analysis showed that hbASCs were enriched for DEGs involved in such BPs as muscle structure development, digestive tract development, tube development, regulation of processes in multicellular organisms, regulation of cell differentiation, regulation of mesenchymal cell proliferation and fat cell differentiation (Figure 5E).

These analyses are consistent with our *in vitro* observations that hbASCs proliferate and differentiate more than haASCs.

### FGF2 acts via PI3K/Akt signaling to regulate hbASC proliferation and adipogenic differentiation

**Figure 6:**
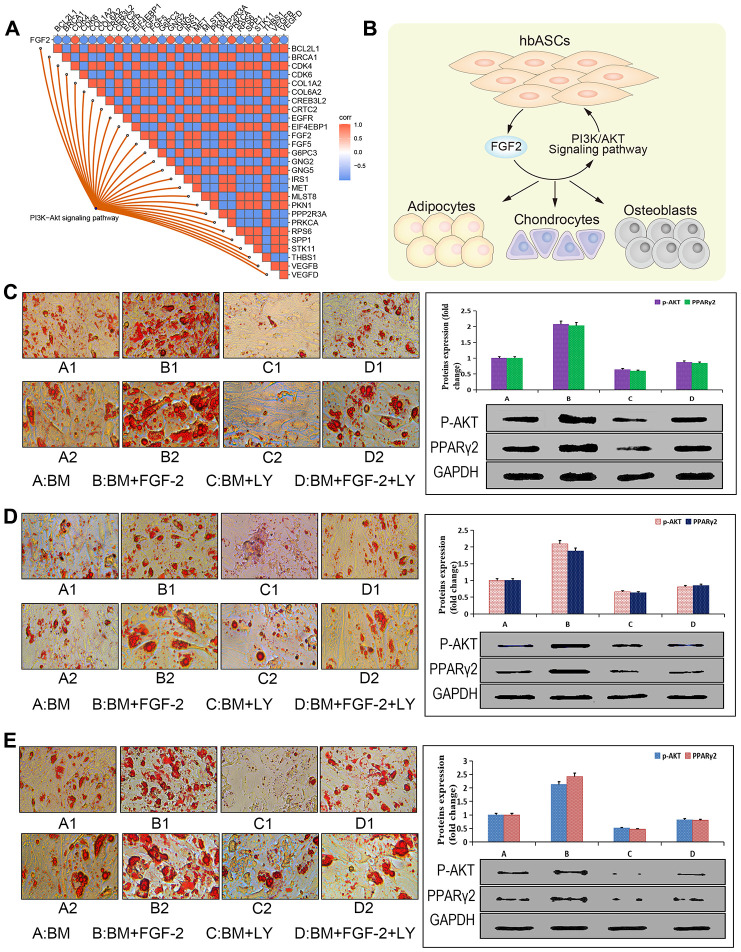
**Promotion of adipogenic differentiation by FGF2 via the PI3K/Akt signaling pathway *in vitro*.** (**A**) Correlation analysis plot of the FGF2-PI3K/Akt signaling pathway. (**B**) Potential mechanism by which this pathway enhances proliferation and differentiation of hbASCs. (**C**) Oil Red O staining of hbASCs showing that FGF2 promoted adipogenic differentiation. (**D**) Oil Red O staining of htASCs showing that FGF2 promoted adipogenic differentiation. (**E**) Oil Red O staining of haASCs showing that FGF2 promoted adipogenic differentiation. Akt and PPARγ2 levels were significantly higher in group B than in groups A, C, or D, based on western blot. LY294002 suppressed adipogenic differentiation of htASCs, haASCs, and hbASCs based on Oil Red O staining, while down-regulating Akt and PPARγ2 based on western blotting. *P<0.01, **P<0.01. Group descriptions: (**A**) basic adipogenic induction medium (BM); (**B**) BM+FGF2 (0.1 μg/mL); (**C**) BM+LY294002; (**D**) BM+FGF2 (0.1 μg/mL)+LY294002. Magnification of panels A1-D1, 200×. Magnification of panels A2-D2, 400×.

Based on the bioinformatics analysis and experiments, we further explored the relationship between FGF2 and PI3K-Akt signaling. FGF2 expression positively correlated with that of genes in the PI3K-Akt signaling pathway (Figure 6A), suggesting that hbASCs may promote their own proliferation and differentiation by secreting FGF2 (Figure 6B). To test this idea, we used Oil Red O staining to compare the numbers of cytoplasmic lipid droplets in the three types of ASCs (Figure 6C–6E): the numbers of droplets varied directly with the level of FGF2. Furthermore, we found that treating the three types of ASCs with the PI3K inhibitor LY294002 reduced the number of cytoplasmic lipid droplets and down-regulated Akt and PPARγ2, while treating cells with exogenous FGF2 exerted the opposite effects. These results suggest that FGF2 promotes adipogenic differentiation via the PI3K/Akt signaling pathway.

## DISCUSSION

Stem cell transplantation and regenerative medicine have shown increasing promise for the replacement of damaged or aging tissue [25–29]. However, stem cells isolated from embryos, umbilical cord blood, or bone marrow can lose quality as they age, they are often difficult to harvest and manipulate, and they are expensive to prepare [30–33]. ASCs, in contrast, can be collected, processed and propagated in a simple, minimally invasive way from excised fat tissue or lipoaspirate. Their pluripotency and proliferative efficiency are similar to those of bone marrow-derived stem cells, and donor morbidity is lower than for MSCs harvested from other sites. Perhaps most importantly, adipose tissue can produce 100-1000 times more MSCs per cubic centimeter than bone marrow [34]. In addition, ASCs survive longer and do not undergo senescence in culture as rapidly as bone marrow-derived stem cells, which allows for greater procedural flexibility. These characteristics suggest that ASCs are well suited to regenerative medicine [35–38], but the best source of ASCs has not yet been elucidated. Our results here suggest that breast may be better than thigh or abdomen as a source of ASCs for regenerative therapy.

Our research described successful isolation of ASCs from the adipose tissue of the abdomen, thigh, and breast, suggesting their potential application in regenerative medicine. Moreover, we found that hbASCs showed greater proliferation and differentiation ability than htASCs or haASCs, perhaps reflecting the differences between breast adipose tissue and other subcutaneous fat deposits in the body. Flow cytometry showed that the proportions of cells positive for CD49d or CD54 were significantly higher in hbASCs than in htASCs or haASCs. CD49d corresponds to the α chain of integrin, while CD54 is a cell adhesion molecule, and both play an important role in cell surface adhesion and signal transmission [39, 40]. Cell adhesion molecules act through various mechanisms to regulate stem cell proliferation, self-renewal, adhesion and multi-lineage differentiation [41]. Therefore, high expression of CD49d and CD54 may help explain the greater differentiation potential of hbASCs.

To further understand why hbASCs show greater proliferation and differentiation potential, we performed comparative RNA-seq. This identified several genes related to paracrine function that were up-regulated in hbASCs relative to haASCs. Consistently, hbASC cultures secreted higher levels of the paracrine factors FGF2, TIMP-1, and SDF-1 than htASCs or haASCs. Consistently, another study showed that breast ASCs express significantly higher levels of FGF2 than ASCs from other sources [42]. In the present study, the genes TIMP-1, CXCR4, FGF2 and FGF9 were up-regulated in hbASCs, while FGF3 and FGF6 were down-regulated. FGF2, one of the best studied fibroblast growth factors, participates in cell division, differentiation, proliferation life, survival, carcinogenesis, and self-renewal of stem cells [43–45]. Our results identify several molecular differences that help explain why hbASCs show greater potential in regenerative medicine than htASCs or haASCs.

Functional enrichment analysis showed that DEGs in hbASCs are involved in MAPK and PI3K/Akt signaling pathways, which are involved in proliferation and differentiation. In particular, the PI3K/Akt signaling pathway has been shown to play an important role in the proliferation and differentiation of ASCs [46, 47]. Given our finding of FGF2 up-regulation in hbASCs, we asked whether this paracrine factor may act via the PI3K/Akt pathway. We found that, indeed, FGF2 levels correlated directly with expression of DEGs involved in the PI3K/Akt signaling pathway, and treatment of ASCs with exogenous FGF2 enhanced their adipogenic differentiation and up-regulated Akt and PPARγ2, while treatment with the PI3K inhibitor LY24402 led to the opposite effects. These results suggest that FGF2 promotes adipocyte differentiation through the PI3K/Akt signaling pathway, which may help explain why hbASCs have stronger differentiation ability than the other two types of ASCs.

Interestingly, hbASCs expressed higher levels of FGF2 protein than haASCs but lower levels of FGF2 mRNA. This may relate to the fact that FGF2 is translated from an internal ribosome entry site on the mRNA [48], and that FGF2 is regulated at the post-transcriptional stage. According to RNA interactome database (http://www.rna-society.org/raid/) [49], we also found that the post-transcriptional inhibitor PUM2 [50], which shows a strong binding score (0.6668) to FGF2 and is down-regulated in hbASCs relative to haASCs (Supplementary Figure 1). We cannot exclude that intrinsic variations in FGF2 expression across individuals may also contribute to the differences that we observed across the three ASC types [51].

Our results should be interpreted with caution in light of several limitations. First, the samples used in this experiment came from plastic surgery, so it was difficult to obtain adipose stem cells from three locations in the same patient. In addition, we did not record donor age or sex, which would be important for comparing the robustness of the three ASC types to aging or hormone exposure. We also did not examine whether adding exogenous FGF2 to ASCs can even out the differences that we observed here, in which case the promising characteristics of hbASCs could also be achieved in ASCs from other sources.

## CONCLUSION

Human ASCs from breast show stronger proliferation and adipogenic differentiation ability than ASCs from thigh or abdomen. This may be due to FGF2 up-regulation, which stimulates the PI3K/Akt signaling pathway.

## MATERIALS AND METHODS

### Patients and samples

Breast fat from five healthy adult donors and abdominal subcutaneous fat from two healthy adult donors were used for RNA sequencing (RNA-seq). In addition, subcutaneous adipose tissue was harvested from abdomen, thigh and breast of 10 women undergoing reduction mammoplasty (Table 2). These procedures were performed with the approval of the Research Ethics Committee of the Fifth Affiliated Hospital of Guangxi Medical University, and all subjects signed consent forms. The volunteers were patients of the Fifth Affiliated Hospital of Guangxi Medical University and the Nanning Dream Plastic and Aesthetic Hospital between March 2015 and June 2017.

**Table 2:** Subject characteristics.

**Subject**	**Age (yr)**	**Sex**	**Ethnicity**	**Locations sampled**	**Height (cm)**	**Weight (kg)**	**Body mass index, kg/m^2^**
**S1**	28	F	Han	Abdomen, Thigh	161	68	26.23
**S2**	32	F	Chuang	Breast	166	71	25.77
**S3**	41	F	Chuang	Abdomen, thigh	159	65	25.71
**S4**	38	F	Yao	Breast	167	75	26.89
**S5**	30	F	Chuang	Thigh	158	66	26.44
**S6**	36	F	Yao	Thigh	157	64	25.96
**S7**	45	F	Han	Thigh	160	70	27.34
**S8**	26	F	Han	Abdomen	163	65	24.46
**S9**	35	F	Miao	Abdomen	162	63	24.01
**S10**	43	F	Han	Breast	165	72	26.45

### Isolation and identification of ASCs

ASCs were isolated and cultured as previously described [52, 53]. First-passage htASCs, haASCs, and hbASCs were analyzed using a Becton Dickinson FACS Calibur flow cytometer. Cells (1×10^6^) were incubated for 30 min with either fluorescein isothiocyanate (FITC)- or phycoerythrin (PE)-conjugated antibodies (BD Biosciences, NJ, USA) against the following ASC surface markers, selected based on previous studies [54–56]: CD13/PE, CD14/FITC, CD29/PE, CD31/FITC, CD34/FITC, CD44/PE, CD45/FITC, CD49d/PE, CD54/PE, CD90/PE, CD105/PE, CD106/FITC, HLA-ABC/FITC, and HLA-DR/FITC. The negative control IgG-PE antibodies were added in the dark at room temperature and incubated for 30 min. Data analysis was performed using Cell Quest Pro acquisition software (BD Biosciences). In order to detect cell differentiation, third-passage (P3) htASCs, haASCs, and hbASCs were cultured in adipogenic, osteogenic, or chondrogenic culture media. Cell preparations were stained with Oil red O to detect adipogenic differentiation, alizarin red to detect osteogenic differentiation, or toluidine blue to detect chondrogenic differentiation.

### RNA extraction and sequencing

P3 ASCs were used for RNA-seq experiments. The purity, concentration and integrity of RNA samples for sequencing were assessed using Nanodrop, qubit 2.0 and agent 2100 methods. Library construction and RNA-seq were performed according to instructions. RNA-seq was performed on an Illumina hiseqtm 2500 in the case of haASCs or on a NovaSeq system in the case of hbASCs. Raw RNA-seq data were filtered to remove low-quality and interrupted reads to obtain high-quality clean data, which were then stored in FASTQ file format. The reads obtained by sequencing each sample of haASCs and hbASCs were compared to the reference genome using BWA software [57].

### Labeling of ASCs with adenovirus-delivered GFP (Ad-GFP)

P3 ASCs were labeled with green fluorescent protein (GFP). A replication-defective recombinant adenovirus encoding GFP (Ad-GFP) in 200 μL of serum-free medium was added to cells in suspension, corresponding to different multiplicities of infection (MOIs) ranging from 0 to 200, and the flask was gently shaken every 15 min for 2 h. Then medium containing 2.5% fetal bovine serum (FBS) was added to the flask. Transduction efficiency was determined by flow cytometry after 48 h. The resulting optimal MOI for transduction of ASCs was used in subsequent steps. The cells were directly analyzed and green autofluorescence was detected by inverted fluorescence microscopy (Leica, Germany). Three days later, P3 htASCs, haASCs, and hbASCs were collected for transplantation *in vivo*.

### Measurement of cell growth and proliferation

Growth and proliferation of P3 htASCs, haASCs, and hbASCs were examined using the cell counting kit-8 (CCK-8) method. The cells were transferred to a 96-well plate at a density of 2×10^4^ cells/well and cultured for 24 h at 37 ºC with 50 mL/L CO_2_. Cells were then randomly divided into 3 groups, with six wells per group (n=6). After 10 additional days of culture, during which the medium was changed at the same time in all groups, 50 μL CCK-8 working fluid was added, and the culture plates were incubated another 4 h. After 10-min oscillations, optical density at 450 nm was determined using a microplate reader in order to obtain growth curves.

### ASC colony formation assays

The three groups htASCs, haASCs, and hbASCs were seeded in 24-well plates at a density of 5000 cells/well and cultured at 37 ºC with 50 mL/L CO2 for 14 days. The cells were then fixed with 100% methanol and stained with 0.1% (w/v) crystal violet. Macroscopic cell colonies were counted in terms of colony forming units (CFU) using Image-Pro Plus 6.0 software (Media Cybernetics, Silver Spring, MD, USA). Each measurement was performed four times. At the same time, cell colonies were counted under a contrast phase microscope prior to staining.

### Paracrine activity of ASCs *in vitro*

The three groups htASCs, haASCs, and hbASCs were seeded in 12-well plates at a density of 2×10^4^ cells/well and cultured at 37 ºC with 50 mL/L CO_2_ for 7 days. After 3 and 7 days, the supernatant was collected for enzyme-linked immunosorbent assay (ELISA) of the following factors: vascular endothelial growth factor (VEGF), fibroblast growth factor-2 (FGF2), epidermal growth factor (EGF), granulocyte-macrophage colony-stimulating factor (GM-CSF), tissue inhibitor of metalloproteinases-1 (TIMP-1), insulin-like growth factor-1 (IGF-1), stromal cell-derived factor-1 (SDF-1), transforming growth factor-beta1 (TGF-β1), and interleukin-10 (IL-10). All assays were performed using commercially available Quantikine Colorimetric Sandwich ELISA kits (R&D Systems, Minneapolis, MN, USA) according to the manufacturer’s instructions.

### Adipogenic differentiation capacity of ASCs *in vitro*

The three groups htASCs, haASCs, and hbASCs were cultured for up to 14 days in 6-well plates at a seeding density of 500 cells/mm^2^ with adipogenic induction medium containing 200 μM indomethacin, 10 μM insulin, 0.5 mM 3-isobutyl-1-methylxanthine, and 1 μM dexamethasone. Adipogenic differentiation capacity was determined by Oil Red O staining according to our previous description [24]. In this method, lipids appear red and nuclei appear pale blue. The adipocyte density for each sample was measured in six different visual fields under the same magnification in a blinded fashion. The cell numbers were normalized to square millimeters. The lipids were extracted from the cells with 100% isopropanol and gentle shaking for 5 min. The concentration of the lipids was measured in triplicate based on absorbance at 510 nm.

### Assessment of the ability of FGF2 to promote differentiation of haASCs, hbASCs and htASCs *in vitro*

The htASCs, haASCs, and hbASCs were each seeded onto one 12-well plate (one 12-well plate for each type of ASC) at a density of 1×10^5^ cells/well and were induced in basic adipogenic induction medium (BM) containing 200 μM indomethacin, 10 μM insulin, 0.5 mM 3-isobutyl-1-methylxanthine, and 1 μM dexamethasone (group A), BM supplemented with 0.1 μg/mL FGF2 (group B), BM supplemented with 1 μg/mL LY294002 (group C), and BM supplemented both with 0.1 μg/mL FGF2 and 1 μg/mL LY294002 (group D). Medium was replaced every 3 days, and the cells were maintained in culture for up to 2 weeks. Adipogenic differentiation was determined by Oil Red O staining. At the same time, cell samples from each group were harvested for western blot analysis. Briefly, the cell pellets were sonicated in extraction buffer, extracts were quantified using the Bio-Rad DC protein assay kit (BioRad, Hercules, CA, USA), then equal amounts of protein were lysed with sodium dodecyl sulfate (SDS) sample buffer and transferred to polyvinylidene difluoride (PVDF) membranes (Millipore, Bedford, MA, USA). Membranes were then blocked with blocking solution (Pierce, Rockford, IL, USA), incubated with primary antibodies against human Akt and human peroxisome proliferator-activated receptor gamma 2 (PPARγ2) (all from Abcam, London, UK), followed by incubation with horseradish-peroxidase (HRP)-conjugated secondary antibody. Enhanced chemiluminescence substrate (Supersignal West Dura Detection System, Pierce) was then used for primary antibody detection. GAPDH was used as a control.

### ASC transplantation *in vivo*

Eighteen nude mice (average weight, 18.0 g ± 3.0 g) served as transplantation models. P3 htASCs, haASCs, and hbASCs were labeled with GFP and cultured in adipogenic differentiation induction medium for 7 days. One collagen type I sponge scaffold (10 mm long, 10 mm wide, 5 mm thick) was put into each culture plate, which were divided into three groups and seeded with third-passage haASCs, htASCs or hbASCs (1×10^7^ cells per well) in growth culture medium. At 24 h later, the collagen scaffold-loaded cell suspensions were injected into each mouse subcutaneously at three locations. Each location randomly received 1.0 mL 1×10^7^ cells/mL htASCs, haASCs, or hbASCs. The mice were fed routinely after transplantation.

### Megascopic measurement and adipogenesis quantitation

At 12 weeks post-transplantation, all transplants were excised from the subcutaneous implantation area and weighed using a standard electronic balance. Each group of grafts was divided into two equal parts. One part was fixed with formalin, embedded in paraffin, stained with hematoxylin and eosin (H&E), and observed with an optical microscope. The other part was used immediately to prepare frozen sections, which were stained with Oil Red O. The two types of preparations were analyzed at the same magnification, and fat cell density was quantitated in 5 fields in a blinded fashion. Cell numbers were normalized to square millimeters. Lipids were extracted from cells with 100% isopropanol and gentle shaking for 5 min. Lipid concentrations were measured in triplicate based on absorbance at 510 nm.

### Real-time qPCR

Total RNA was extracted using Trizol reagent (Invitrogen) and treated with DNase I. The cDNA conversion was accomplished using the RevertAid™ First Strand Synthesis Kit (Fermentas), and qPCR was performed using the SYBR® Green PCR Master Mixes on a StepOnePlus Real-Time PCR System (Applied Biosystems) utilizing the primer pairs shown in Table 3. The genes analyzed encode factors related to paracrine function and adipogenesis. The 2^–ΔΔCT^ method [58–60] was used to quantify gene expression relative to that of GAPDH expression. Data were presented as fold change relative to the control.

**Table 3:** qPCR primer sequences for target genes.

**Gene**	**Forward**	**Reverse**	**Accession ID**
**TIMP-1**	CTTCTGCAATTCCGACCTCGT	AAGTATCCGCAGACACTCTCC	NM_001044384
**CXCR4**	CTTCATCAGTCTGGACCGCTA	CATCTGCCTCACTGACGTTG	XM_006529113
**FGF-1**	TTCACAGCCCTGACCGAGA	TATAAAAGCCCGTCGGTGTC	XM_006525647
**FGF-2**	GCGACCCTCACATCAAGCTAC	AAGAAACACTCATCCGTAACACA	XM_903202
**FGF-3**	ACCTCCACTGCCGTTATCTCC	GCAAGCTCTACTGCGCCACGAA	NM_008007
**FGF-4**	ACGAAGCCAATATGTTAAGTGT	TTATTCAGGGCCACATACCAC	NM_010202
**FGF-5**	CAAAGTCAATGGATCCCACGAA	GTCATCTGTGAACTTGGCACT	NM_001277268
**FGF-6**	GTGCCCTCTTCGTTGCCAT	GCTTTACCCGTCCGTATTTGCT	NM_010204
**FGF-7**	TGACTCCAGAGCAAATGGCTA	TTTGATTGCCACAATTCCAAC	NM_008008
**FGF-8**	ACCAACTCTACAGCCGCACCA	ACAATCTCCGTGAAGACGCAGT	NM_001166361
**FGF-9**	CTTCCCCAATGGTACTATCCAG	ATTCATCCCGAGGTAGAGTCCA	NM_013518
**FGF-10**	TGCGGAGCTACAATCACCT	TGACGGCAACAACTCCGAT	NM_008002
**PPARγ2**	TGTCTCATAATGCCATCAGGT	TCTTTCCTGTCAAGATCGC	XM_006505743
**C/EBPα**	GCCGCGCACCCCGACCTCC	CCCCGCAGCGTGTCCAGTTCG	NM_001287514
**ADD1**	AGTACAAAGCCAAGTCCCGTTC	CCCGAATCACCGTCACTAGCAA	NM_001024458
**FAD24**	GGGAACTTGAGGAAGAGATCATTG	GGATCTGATAATATGGCAGATGCC	NM_021315
**GAPDH**	CAAATTCCATGGCACCGTCA	GACTCCACGACGTACTCAGC	NM_001289726

### Analysis of differential expression and functional enrichment

Genes differentially expressed (DEGs) between hbASCs and haASCs were screened using the *limma* package [61] in R. Genes with P < 0.05 and |log_2_ fold change (FC)| > 1 were considered to be DEGs. The subset of DEGs associated with a P (adjusted by the false discovery rate) < 0.01 and |log_2_ FC|> 2 were selected to perform Gene Ontology (GO) and Kyoto Encyclopedia of Genes and Genomes (KEGG) pathway enrichment analyses using the *clusterProfiler* package [62] in R, for which P < 0.05 was considered significant.

### Gene set enrichment analysis (GSEA) and ClueGO analysis

GSEA was performed using the normalized gene expression profiles to explore biological processes (BP) and Kyoto Encyclopedia of Genes and Genomes (KEGG) pathways related to ASCs. The Java software of GSEA (version 2-2.2.4) was used in the analysis. The c5.bp.v6.2.symbols.gmt and c2.cp.kegg.v6.2.symbols.gmt datasets in the MsigDB V6.2 database [63] were used as reference gene sets, and GSEA was performed with default parameters. In addition, ClueGO [64] in Cytoscape [65] was used to analyze BP enrichment for selected DEGs.

### Statistical analysis

Data were reported as mean ± standard deviation (SD). We performed an analysis of variance to determine whether the means of all groups were similar. This approach took into account intra- and inter-group variation. Furthermore, if the analysis of the variance of the three means revealed statistically significant differences, all pairs of means were compared using paired t-tests. Differences between means were regarded as significant if the resulting two-tailed P was < 0.05. All data were analyzed using SPSS for Windows 17.0 (Chicago, IL, USA).

## Supplementary Material

Supplementary Figure 1
