# Supplementary material for: FGF2-induced PI3K/Akt signaling evokes greater proliferation and adipogenic differentiation of human adipose stem cells from breast than from abdomen or thigh
Source: Aging (Albany NY). 2020 Jul 24;12(14):14830–48. doi: 10.18632/aging.103547 (PMC7425436; doi:10.18632/aging.103547)
Supplement: Supplementary Figure 1 [file aging-12-103547-s001..pdf]

SUPPLEMENTARY FIGURE

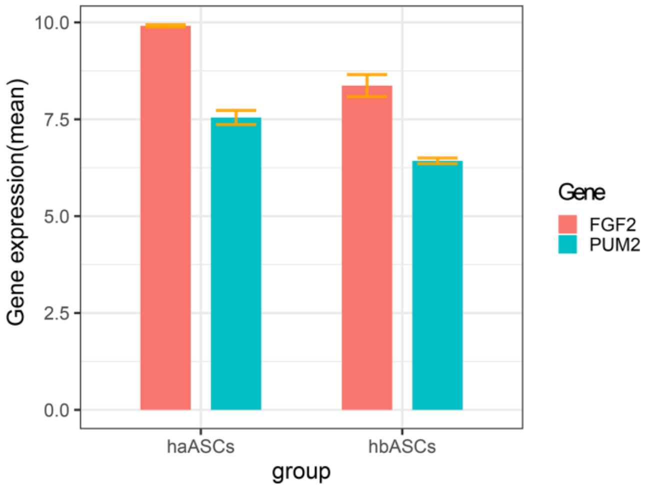

Supplementary Figure 1. Gene expression of FGF2 and PUM2 in haASCs and hbASCs.
